# Influence of enhanced recovery after surgery (ERAS) on patients receiving lung resection: a retrospective study of 1749 cases

**DOI:** 10.1186/s12893-020-00960-z

**Published:** 2021-03-06

**Authors:** Chunmei Wang, Yutian Lai, Pengfei Li, Jianhuan Su, Guowei Che

**Affiliations:** 1 Department of Thoracic Surgery, West China Hospital, Sichuan University, Chengdu, 610041 People’s Republic of China; 2 Rehabilitation Department, West China Hospital, Sichuan University, Chengdu, 610041 People’s Republic of China; 3 Lung Cancer Center, West China Hospital, Sichuan University, Chengdu, 610041 People’s Republic of China

**Keywords:** Enhanced recovery after surgery, Lung cancer, Postoperative pulmonary complications, Thoracic surgery

## Abstract

**Background:**

The study aimed to evaluate the outcomes following the implementation of enhanced recovery after surgery (ERAS) for patients undergoing lung cancer surgery.

**Method:**

A retrospective cohort study involving 1749 patients with lung cancer undergoing pulmonary resection was conducted. The patients were divided into two time period groups for analysis (routine pathway and ERAS pathway). Logistic regression analysis was performed to assess the risks of developing postoperative pulmonary complications.

**Results:**

Among the 1749 patients, 691 were stratified into the ERAS group, and 1058 in to the routine group. The ERAS group presented with shorter postoperative in-hospital length of stay (LOS) (4.0 vs 6.0, P < 0.001), total LOS (10.0 vs. 13.0 days, P < 0.001), and lower total in-hospital costs (P < 0.001), including material (P < 0.001) and drug expenses (P < 0.001). Furthermore, the ERAS group also presented with a lower occurrence of postoperative pulmonary complications (PPCs) than the routine group (15.2% vs. 19.5%, P = 0.022). Likewise, a significantly lower occurrence of pneumonia (8.4% vs. 14.2%, P < 0.001) and atelectasis (5.9% vs. 9.8%, P = 0.004) was found in the ERAS group. Regarding the binary logistic regression, the ERAS intervention was the sole independent factor for the occurrence of PPCs (OR: 0.601, 95% CI 0.434–0.824, P = 0.002). In addition, age (OR: 1.032, 95% CI 1.018–1.046), COPD (OR: 1.792, 95% CI 1.196–2.686), and FEV1 (OR: 0.205, 95% CI 0.125–0.339) were also independent predictors of PPCs.

**Conclusion:**

Implementation of an ERAS pathway shows improved postoperative outcomes, including shortened LOS, lower in-hospital costs, and reduced occurrence of PPCs, providing benefits to the postoperative recovery of patients with lung cancer undergoing surgical treatment.

## Background

Thoracic surgery is the optimal therapy for early or resectable lung cancer [1, 2]; however, it remains an invasive and traumatic procedure for the patients, particularly for those with lung dysfunction or a poor health status [3]. Minimally invasive surgeries, including video-assisted thoracoscopic surgery (VATS), are preferred for early stage lung cancer but remain limited by side effects and surgical stress, with a morbidity of 20.8–34.1% after lung cancer surgery [4, 5]. The search for better recovery from surgical injury is an urgent issue for lung cancer therapy.

Recently, researchers have begun to pay increasing attention to patient recovery after surgery [6, 7]. Enhanced recovery after surgery (ERAS), an evidence-based multimodal protocol for perioperative care proposed in colorectal surgery in the late 1990s, combines various synergistic elements throughout the hospital stay, from the first consultation to discharge. ERAS strategies have been considerably developed and enrolled a growing number of elements in all aspects of perioperative care, aiming to largely reduce the stress response to surgery, complications, and the in-hospital length of stay (LOS) after surgery. Great effort has been made to improve or optimize perioperative management.

A series of studies have focused on ERAS strategies for thoracic surgery in the past few years, indicating that ERAS can efficiently minimize the surgical trauma, reduce postoperative complications, improve quality of life before and after surgery, shorten hospital stay and finally decrease the financial burden of patient [8–11]. The research of ERAS still needs to be studied and refined [12–16]. A larger sample size and more diverse regions of study are needed to determine the effectiveness of ERAS strategies.

For this reason, we conducted this study to evaluate the clinical outcomes of an ERAS program among patients with cancer after anatomical lung resection.

## Method

The ERAS protocol was developed and implemented in the thoracic department of our hospital following approval from the China International Exchange and Promotion Association for Medical and Healthcare (CMAP) as the training center for ERAS teaching and practices. The ERAS team, consisting of surgeons, physical therapists, nutritionists, specialized nurses and anesthesiologists, was created in 2015.

### Patients

The patients were divided into two time period groups for analysis (routine pathway: 2012.01.01–2014.12.31 and ERAS pathway: 2016.01.01–2017.12.31). A washout period of 1 year was set to diminish the impact of carryover effects. The clinical outcomes between the two groups were comparatively analyzed. The inclusion criteria were as follows: (1) diagnosis with primary non-small-cell lung cancer (NSCLC); (2) anatomical lung resection, including lobectomy and segmentectomy; and (3) age ≥ 18 years old. Exclusive criteria included (1) benign lesions or other pathological types except NSCLC; (2) other surgical approaches except anatomical lung resection; and (3) age < 18 years old. A total of 1058 patients who underwent anatomical lung resection in our department between 2012.01.01 and 2014.12.31 were selected. During this period, patients underwent the routine pathway and were enrolled into the pre-ERAS group; 691 subjects included between 2016.01.01 and 2017.12.31 and who underwent the standard ERAS pathway were classified into the ERAS group.

### Comparison of the ERAS program and the routine pathway

The ERAS program standardizes various the elements throughout hospitalization. The whole ERAS program implemented in this study included ERAS education and consultation, venous thromboembolisms (VTEs) prophylaxis, chest tubes, urinary catheters, postoperative pain and nutrition management, anesthesia, perioperative respiratory training, and mobilization. During respiratory training and mobilization, patients were encouraged to finish 20 breaths/session for 3 sessions/day via a volumetric incentive spirometer (HUDSON RCI 2500, TeleflexInc, USA) perioperatively, under the guidance and supervision of physical therapists. Moreover, a specialist nurse recorded the patient performance. The nurse of the VTE management group evaluated the VTE risk via the Caprini Assessment Scale [17, 18] and used low-molecular-weight heparin (LMWH) perioperatively for VTE prophylaxis [19]. According to the Caprini score, patients were divided into low-, moderate- and high-risk groups. The low- and moderate-risk groups received enoxaparin (4000 Axa IU, q.d) from referral to discharge, while the high-risk group received enoxaparin (4000 AxaIU, q.d) from referral to 2–4 weeks after discharge. Coagulation function was monitored. For the pre-ERAS group, patients did not receive a Caprini score or standardized VTE prophylaxis. The specialist nurse supervised and assessed the postoperative pain intensity (NRS score* 6, 12, 18, 24, 36, 48 h after surgery) and thereby adjusted the analgesics if necessary. Postoperative analgesia was assessed by a standard protocol-based multimodal approach: acetaminophen 1000 mg i.v. × 48 h, then p.o., ketorolac 15 mg i.v. × 48 h, then NSAIDS p.o., and gabapentin 300 mg p.o. Breakthrough pain was treated as follows: tramadol 50 mg p.o., then oral or i.v. opioids if necessary (sustained pain score 4/10). Other elements of the ERAS program are listed in Table 1.

**Table 1:** Comparison of Pre-ERAS (routine) pathway and ERAS pathway

Pre-ERAS (routine) pathway	ERAS pathway
General patient admission education introduced by video	The content included general patient admission education, instruction about ERAS principles and ERAS member responses to the patients. The specialized nurse was mainly responsible for the session. Smoking and alcohol cessation for 2 weeks and 4 weeks respectively
Perioperative respiratory training and mobilization	
No	A volumetric Incentive spirometer (HUDSON RCI 2500, TeleflexInc, USA) was provided after the agreement of the patients. The physical therapists would teach the patients and supervised the patients’ respiratory training during the in-hospital period On the first day after the operation, the electrocardiograph monitoring will be withdrawn and the patient will get out of bed for activities under professional escort. If the patient cannot get out of bed due to pain or anesthesia, the patient will also be encouraged to sit up or stand up beside the bed
VTE management	
No standardized management	1. VTE prophylaxis education preoperative VTE assessment risk rating 2. LMWH use for VTE prophylaxis during perioperative period 3. Early mobilization after surgery
Chest tube management	
1. Traditionally using 2 28-F chest tube 2. Chest tube removal after drainage volume < 200 ml	1. Small-bore chest tube (18-Floyle) 2. Drain removed < 400 ml/24 h and no air flow
Urinary catheter management	
Urinary catheter was routinely used	No urinary catheter
Postoperative pain management	
Non-standardized management	1. Specialized nurse supervised and assess the pain intensity (NRS score at 6 h, 12 h, 18 h, 24 h, 36 h, 48 h after the surgery), and based on it, adjustment of analgesics would be performed if necessary 2. Standardized protocol-based multimodal approach to post-operative analgesia 3. Specialized nurses open thoracic pain clinics, aiming at follow-up of postoperative pain
Postoperative nutrition management	
Non-standardized management	1. Early Oral Intake 2. Medium chain triglyceride (MCT) diet supported by nutriology department

*VTE* venous thromboembolism; *LMWH* low-molecular-weight heparin, *NRS* numeric rating scales, *MCT* medium chain triglyceride

### Data collection and statistical analysis

Patient demographics, comorbidities, and clinical outcomes during hospitalization, including length of stay, readmissions, reoperations, and postoperative pulmonary complications (PPCs), were retrospectively recorded in both the ERAS and pre-ERAS groups. Ten types of criteria for PPCs were investigated on the basis of the STS/ESTS definitions [20]: (1) pneumonia; (2) atelectasis documented clinically or radiographically; (3) respiratory/heart failure or adult breathing distress syndrome (ADRS); (4) mechanical ventilation > 48 h; (5) > 7 days air leak; (6) pulmonary embolism; (7) empyema; (8) chyle leak; (9) bronchopleural fistula; and (10) return to the ICU.

Continuous variables are expressed as the means and standard deviations (SDs). Data that did not follow a normal distribution are presented as the median and range, and binary variables are presented as proportions. Data were evaluated via the chi-square test, Fisher’s exact test, or Student’s t-test as appropriate. Statistical analyses were performed on SPSS 21.0 at the significance level of 0.05.

## Results

### Clinical characteristics

The 1749 patients were divided into an ERAS group (n = 691) and a routine group (n = 1058). Clinical data including age, sex, comorbidities, pathologic stage and type, pulmonary function, and amount of bleeding during the operation were compared between groups (Table 2). Compared with the routine pathway group, the ERAS group was found to have a higher proportion of VATS (74.2% vs. 65.3%), shorter postoperative in-hospital LOS [4.0 (2.0, 6.0) vs. 6.0 (4.0, 9.0)], shorter total LOS [10.0 (7.0, 13.0) vs. 13.0 (10.0, 16.0) days], and lower total in-hospital costs, including material and drug expenses, all with significant differences (all P < 0.001). Regarding to drainage, a lower drainage volume [540.0 (275.0, 1170.5) vs. 700.0 (150, 1710) ml] and a shorter duration of drainage [3.0 (2.0, 5.0) vs. 5.0 (4.0, 7.0) days] were observed in the ERAS group (both P < 0.001).

**Table 2:** Clinical characteristic between two groups

	ERAS group n = 691	Routine group n = 1058	P
Basic clinical characteristics			
Age (year)^a^	61.0 (56.0, 67.0)	61.0 (53.0, 68.0)	0.688
Sex (M/F)	351/340	527/531	0.687
Comorbidities			
Hypertension (Y/N)	111/580	151/907	0.305
COPD (Y/N)	71/620	97/961	0.443
Diabetes mellitus (Y/N)	62/629	88/970	0.633
Smoking status (Y/N)	182/509	249/809	0.595
Pulmonary function			
FEV1 (L)^a^	2.29 (1.71, 2.73)	2.28 (2.12, 2.44)	0.807
FVC (L)^a^	3.26 (2.72, 3.71)	3.20 (2.94, 3.48)	0.169
Surgical approach (n %)			
VATS	513 (74.2)	691 (65.3)	< 0.001
Open	178 (25.8)	367 (34.7)	
Amount of bleeding in operation^a^	50.0 (20.0, 70.0)	180.0 (140.0, 220.0)	0.628
Surgery time^a^	90.0 (70.0, 120.0)	120.0 (90.0, 150.0)	< 0.001
Postoperative LOS^a^	4.0 (2.0,6.0)	6.0 (4.0, 9.0)	< 0.001
Total LOS^a^	10.0 (7.0, 13.0)	13.0 (10.0, 16.0)	< 0.001
Pathological stage, (n %)			
Stage I	313 (45.3)	506 (47.8)	0.589
Stage II	292 (42.3)	421 (39.8)	
Stage III	75 (10.9)	119 (11.2)	
Stage IV	11 (1.6)	12 (1.1)	
Pathological type (n %)			
Adenocarcinoma	473 (68.5)	740 (69.9)	0.799
Squamous cell carcinoma	192 (27.8)	281 (26.6)	
Other	26 (3.8)	37 (3.5)	
In-hospital expense, ¥			
Total^a^	46,047.7 (39,068.7, 52,733.8)	47,583.0 (43,761.6, 51,839.6)	< 0.001
Material cost^a^	23,742.6 (19,588.2, 27,844.8)	25,040.4 (21,439.5,29,871.5)	< 0.001
Drug cost^a^	7633.5 (5537.0, 10,100.3)	8157.6 (6453.2, 11,665.5)	< 0.001
Drainage volume^a^	540.0 (275.0, 1170.5)	700.0 (150, 1710)	< 0.001
Duration of drainage^a^	3.0 (2.0, 5.0)	5.0 (4.0, 7.0)	< 0.001

*ERAS* enhanced recovery after surgery, *COPD* chronic obstructive pulmonary disease defined as FEV1/FVC < 70% and FEV1 < 80% of predicted, *FEV1* forced expiratory volume in 1 s, *FVC* forced vital capacity, *VATS* video assisted thoracic surgery, *LOS* length of stay

^a^Non-normal distribution variables described as median (interquartile)

^¥^China Yuan

### Occurrence of PPCs

Compared with the routine pathway group, the ERAS group presented with a lower occurrence of PPCs (15.2%, 105/691 vs. 19.5%, 206/1058, P = 0.022) and a significantly lower occurrence of pneumonia (8.4%, 58/691 vs. 14.2%, 150/1058, P < 0.001) or atelectasis (5.9%, 41/691 vs. 9.8%, 104/1058, P = 0.004) (Table 3).

**Table 3:** Postoperative pulmonary complications between the two groups

	ERAS group n = 691	Routine group n = 1058	P value
PPCs rate, (n %)	105 (15.2)	206 (19.5)	0.022
Pneumonia	58 (8.4)	150 (14.2)	< 0.001
Atelectasis	41 (5.9)	104 (9.8)	0.004
Pulmonary embolism	2 (0.3)	4 (0.4)	1.000
Respiratory/heart failure or ADRS	11 (1.6)	12 (1.1)	0.411
Bronchopleural fistula	6 (0.9)	10 (0.9)	0.869
Mechanical ventilation > 48 h	18 (2.6)	35 (3.3)	0.239
Empyema	4 (0.6)	11 (1.0)	0.307
Air leak	35 (5.1)	78 (7.4)	0.055
Back to ICU	7 (1.0)	11 (1.0)	0.957
Death	4 (0.6)	6 (0.6)	1.000

*PPCs* postoperative pulmonary complications, *ERAS* enhanced recovery after surgery

We also compared clinical characteristics between patients with (n = 311) and without PPCs (n = 1438). Significant differences between groups were found in age, FEV1, amount of bleeding during the operation, COPD, total or postoperative LOS, total in-hospital costs, drug costs, duration of drainage (all P < 0.001), FVC (P = 0.043), and surgery time (P = 0.021) (Table 4).

**Table 4:** Comparison between PPCs group and non-PPCs group

	PPCs group n = 311	Non-PPCs group n = 1438	P
Basic clinical characteristics			
Age (year)^a^	62.0 (56.0, 73.0)	60.0 (54.0, 66.0)	< 0.001
Sex (M/F)	148/163	730/708	0.310
Comorbidities			
Hypertension (Y/N)	48/263	214/1224	0.805
COPD (Y/N)	46/265	122/1316	0.001
Diabetes mellitus (Y/N)	27/284	123/1315	0.942
Smoking status (Y/N)	72/239	370/1068	0.343
ERAS program (Y/N)	105/206	852/586	0.022
Pathological stage I or 0, (Y/N)	140/171	679/759	0.480
VATS approach	203/108	431/1001	0.134
Pulmonary function			
FEV1 (L)^a^	2.13 (1.90, 2.31)	2.32 (2.09, 2.52)	< 0.001
FVC (L)^a^	3.20 (2.84, 3.44)	3.23 (2.87 3.56)	0.043
Amount of bleeding in operation^a^	150.0 (100.0, 200.0)	50.0 (150.0, 200.0)	0.022
Surgery time^a^	120.0 (90.0, 140.0)	110.0 (80.0, 140.0)	0.021
Postoperative LOS^a^	9.0 (6.0,12.0)	4.0 (3.0, 7.0)	< 0.001
Total LOS^a^	16.0 (12.0, 19.0)	8.0 (11.0, 14.0)	< 0.001
In-hospital expense, ¥			
Total^a^	52,649.6 (46,815.1, 61,097.0)	46,151.2 (41,919.2, 50,908.2)	< 0.001
Material cost^a^	24,567.0 (19,886.2, 31,942.8)	24,486.5 (20,925.8, 28,342.1)	0.085
Drug cost^a^	14,411.3 (11,127.8, 16,427.8)	7322.0 (5980.1, 9655.5)	< 0.001
Drainage volume^a^	700 (230.0, 1600.0)	590.0 (180, 1470)	0.095
Duration of drainage^a^	5.0 (3.0, 8.0)	4.0 (3.0, 6.0)	< 0.001

*PPCs* postoperative pulmonary complications, *ERAS* enhanced recovery after surgery, *COPD* chronic obstructive pulmonary disease defined as FEV1/FVC < 70% and FEV1 < 80% of predicted, *FEV1* forced expiratory volume in 1 s, *FVC* forced vital capacity, *VATS* video assisted thoracic surgery, *LOS* length of stay

^a^Non-normal distribution variables described as median (interquartile)

^¥^China Yuan

The relevant independent factors for PPCs, pneumonia or atelectasis were investigated via binary logistic regression. The variables analyzed included sex, age, smoking status, comorbidities, FEV1, FVC, surgical approach, amount of bleeding during the operation, surgery time, and the presence of early stage (stage I) cancer. The ERAS intervention was found to be an independent factor for the occurrence of PPCs (OR: 0.601, 95% CI 0.434–0.824, P = 0.002), pneumonia (OR: 0.371, 95% CI 0.243–0.566, P < 0.001), and atelectasis (OR: 0.431, 95% CI 0.271–0.687, P < 0.001) (Table 5). As listed in Table 5, ERAS intervention, age, COPD, and FEV1 were independent predictors for PPCs.

**Table 5:** Multivariable analysis (n = 101) of risk to postoperative pulmonary complications (PPCs)^a^, pneumonia and atelectasis

Variables	OR (95% CI)	P value
PPCs		
ERAS intervention	0.601 (0.434–0.824)	0.002
Age	1.032 (1.018–1.046)	< 0.001
COPD	1.792 (1.196–2.686)	0.005
FEV1	0.318 (0.221–0.457)	< 0.001
Pneumonia		
ERAS intervention	0.371 (0.243–0.566)	< 0.001
Age	1.039 (1.022–1.055)	< 0.001
FEV1	0.205 (0.125–0.339)	< 0.001
Atelectasis		
ERAS intervention	0.431 (0.271–0.687)	< 0.001
Early stage	0.676 (0.469–0.975)	0.036
FEV1	0.272 (0.157–0.471)	< 0.001

*PPCs* postoperative pulmonary complications

^a^The multivariable analysis was performed via binary logistic regression

## Discussion

Lung cancer continues to ranks first among all cancers in terms of incidence and death rates worldwide, including in China. Surgery is deemed the optimal strategy or option for patients with early or resectable tumors. Due to poor lung function and sequential PPCs, patients with lung cancer often require long hospitalizations and high postoperative costs [21, 22]. Recently, growing attention has been paid to the implementation of ERAS programs [23–26], which are effective in decreasing postoperative morbidity and mortality and were formerly known as “fast-track surgery” introduced by Kehlet and Mogensen in 1999 [27]. ERAS was first proposed for colorectal surgery and has demonstrated clinical benefits in other surgeries in decreasing morbidity, hospital stay, and costs [23–26].

Some issues should be noted before the implementation of an ERAS protocol in lung cancer surgery. (1) Advances in radiography and prevalent cancer screening programs have considerably increased the probability of early detection and of timely surgical therapy. (2) VATS, which significantly alters thoracic operational steps and brings considerable benefits, such as less wound pain and shorter hospital stay, has become the mainstream approach for lung cancer surgery, especially at early-stages [28–30]. (3) The components of ERAS programs vary among different institutions, and the practice of ERAS elements likely relies on clinical experience. Many elements of ERAS programs have become routine and it is difficult to judge whether temporal changes in practice can improve the outcomes, rather than the use of the ERAS pathway per se.

Our ERAS multidisciplinary and collaborative team was established in 2015 to more professionally and effectively carry out the ERAS pathway. The patients are counselled and supervised by trained nurses to complete the ERAS phases. Breathing exercise and postoperative nutrition procedures are conducted by specialized physical therapists and nutritionists.

We found that the proportion of patients who underwent the ERAS pathway with early-stage (stage 0 or I) cancer were not significantly greater than those of patients who underwent the routine pathway, but the proportion of VATS among ERAS patients increased. The possible reasons were that the wide promotion of minimally invasive surgery and the commonly accepted or recognized advantaged of VATS, especially for young surgeons. From the surgeons’ perspective, the implementation of minimally invasive surgery is also an important element of the ERAS protocol, as it offers patients a shorter surgical time, less intraoperative blood loss, postoperative pain and surgical trauma, and faster sequential recovery after surgery. In the guidelines for ERAS drafted by the ERAS Society and the European Society of Thoracic Surgeons (ESTS), a VATS approach for lung resection is recommended for early-stage lung cancer with a high evidence level and strong recommendation grade [26]. According to the results, lower surgery time and less blood loss were observed in the ERAS group, showing the potential benefits that this minimally invasive approach provided to the patients’ postoperative recovery.

PPCs are considered important negative influences on recovery outcomes, increasing the risk of mortality. Evidence shows that ERAS regimens integrating effective perioperative courses prevent PPCs for patients with lung cancer undergoing lung resection [31]. Controversially, Brunelli et al. reported no significant difference in postoperative morbidity after the use of an ERAS program. Potential reasons include the lack of a washout period, study heterogeneity, the exact structure of the ERAS program, and the quality of implementation or patient selection [32]. What’s more, their conventional care was very similar to ERAS before they introduced ERAS. We found lower occurrences of PPCs and pneumonia in the ERAS group than in the routine pathway group. Theoretically, elements including the VATS approach, pain, and VTE management may jointly improve postoperative recovery and decrease the PPC rate. Furthermore, the results of multivariable analysis revealed that the ERAS intervention was an independent factor of PPCs as well as of pneumonia and atelectasis, validating its effectiveness in improving postoperative recovery for those patients. Another essential variable was LOS. Proper pain control, chest tube removal and few complications contribute to a shorter LOS, indicating better postoperative recovery. Early mobilization is the most important predictor of reduced morbidity [11]. One recent systematic review summarized RCTs concerning ERAS and reported that four of the five RCTs indicated the mean LOS was significantly shortened by the ERAS [33]. Our study reveals shorter LOS and postoperative LOS in ERAS group, suggesting better recovery in those population. Meanwhile, lower in-hospital expenses including drug costs and a shorter duration for the indwelling chest tube were found in the ERAS group, which also provided evidence of the effectiveness of the ERAS program.

In the present study, we also explored the predictive factors for developing PPCs. In addition to the ERAS intervention, age, COPD, and FEV1 can also significantly and independently predict the risk of developing PPCs.

The study has some limitations that should not be ignored. First, all the patients were selected from a single regional center by a small group of surgeons, and propensity-matching was not analyzed in the control group. As a retrospective study, the lack of randomization limited the control of intergroup bias. We enrolled the patients over a large time span of approximately 5 years. Therefore, better outcome for the ERAS group may be the result of the bias caused by the increased experience of our team. Second, the effects of the ERAS program, the sole effects of standardization, and whether temporal changes in practice improved the outcomes, rather than the use of the ERAS pathway per se cannot be easily determined. Third, the selection of patients receiving anatomical resection and the exclusion of patients undergoing wedge section and pneumonectomy resulted in a relevant bias and the sequential limitation of generalization of the conclusions. Moreover, we did not detail the in-hospital costs, so we cannot fully explore the economic outcomes of the ERAS program. Finally, we did not assess the pain control and nutrition-related variables between groups, and could not directly assess the role of pain and nutrition management in the ERAS program.

## Conclusion

The use of an ERAS pathway is associated with improved postoperative outcomes, including a shorter LOS and a lower occurrence of PPCs, providing benefits of postoperative recovery for patients with lung cancer undergoing surgical treatment.

## Data Availability

The datasets used and/or analysed during the current study are available from the corresponding author on reasonable request.

## Abbreviations

VATS: Video-assisted thoracoscopic surgery
ERAS: Enhanced recovery after surgery
NSCLC: Non-small-cell lung cancer
VTE: Venous thromboembolism
LMWH: Low-molecular-weight heparin
NRS: Numeric rating scales
PPCs: Postoperative pulmonary complications
SD: Standard deviations
LOS: Length of stay
COPD: Chronic obstructive pulmonary disease
FEV1: Forced expiratory volume in 1 s
FVC: Forced vital capacity

## References

[1] Ghanem S, El Bitar S, Hossri S (2017). What we know about surgical therapy in early-stage non-small-cell lung cancer: a guide for the medical oncologist. Cancer Manag Res.

[2] Sherwood JT, Brock MV (2007). Lung cancer: new surgical approaches. Respirology.

[3] Boffa DJ, Allen MS, Grab JD (2008). Data from The Society of Thoracic Surgeons General Thoracic Surgery database: the surgical management of primary lung tumors. J Thorac Cardiovasc Surg.

[4] Thomas PA, Berbis J, Falcoz PE, EPITHOR Group (2014). National perioperative outcomes of pulmonary lobectomy for cancer: the influence of nutritional status. Eur J Cardiothorac Surg.

[5] Ha D, Choi H, Zell K (2015). Association of impaired heart rate recovery with cardiopulmonary complications after lung cancer resection surgery. J Thorac Cardiovasc Surg.

[6] Nicholson A, Lowe MC, Parker J (2014). Systematic review and meta-analysis of enhanced recovery programmes in surgical patients. Br J Surg.

[7] Lee L, Mata J, Ghitulescu GA (2015). Cost-effectiveness of enhanced recovery versus conventional perioperative management for colorectal surgery. Ann Surg.

[8] Joliat GR, Labgaa I, Petermann D (2015). Cost benefit analysis of an enhanced recovery protocol for pancreaticoduodenectomy. Br J Surg.

[9] Roulin D, Donadini A, Gander S (2013). Cost-effectiveness of the implementation of an enhanced recovery protocol for colorectal surgery. Br J Surg.

[10] Senturk JC, Kristo G, Gold J (2017). The development of enhanced recovery after surgery across surgical specialties. J Laparoendosc Adv Surg Tech A.

[11] Rogers LJ, Bleetman D, Messenger DE (2018). The impact of enhanced recovery after surgery (ERAS) protocol compliance on morbidity from resection for primary lung cancer. J Thorac Cardiovasc Surg.

[12] Das-Neves-Pereira JC, Bagan P, Coimbra-Israel AP, Grimaillof-Junior A, Cesar-Lopez G, Milanez-de-Campos JR (2009). Fast-track rehabilitation for lung cancer lobectomy: a five-year experience. Eur J Cardiothorac Surg.

[13] Salati M, Brunelli A, Xiumè F, Refai M, Pompili C, Sabbatini A (2012). Does fast-tracking increase the readmission rate after pulmonary resection? A case-matched study. Eur J Cardiothorac Surg.

[14] Khandhar SJ, Schatz CL, Collins DT, Graling PR, Rosner CM, Mahajan AK (2018). Thoracic enhanced recovery with ambulation after surgery: a 6-year experience. Eur J Cardiothorac Surg.

[15] Madani A, Fiore JF, Wang Y, Bejjani J, Sivakumaran L, Mata J (2015). An enhanced recovery pathway reduces duration of stay and complications after open pulmonary lobectomy. Surgery.

[16] Fiore JF, Bejjani J, Conrad K, Niculiseanu P, Landry T, Lee L (2016). Systematic review of the influence of enhanced recovery pathways in elective lung resection. J Thorac Cardiovasc Surg.

[17] Obi A, Pannucci CJ, Nackashi A (2015). Validation of the caprini venous thromboembolism risk assessment model in critically ill surgical patients. JAMA Surg.

[18] Fu Y, Liu Y, Chen S (2018). The combination of Caprini risk assessment scale and thrombotic biomarkers to evaluate the risk of venous thromboembolism in critically ill patients. Medicine (Baltimore).

[19] Van Dongen CJ, MacGillavry MR, Prins MH (2005). Once versus twice daily LMWH for the initial treatment of venous thromboembolism. Cochrane Database Syst Rev.

[20] Seder CW, Salati M, Kozower BD (2016). Variation in pulmonary resection practices between the society of thoracic surgeons and the European society of thoracic surgeons general thoracic surgery databases. Ann Thorac Surg.

[21] Lugg ST, Agostini PJ, Tikka T (2016). Long-term impact of developing a postoperative pulmonary complication after lung surgery. Thorax.

[22] Pearse RM, Moreno RP, Bauer P (2012). Mortality after surgery in Europe: a 7 day cohort study. Lancet.

[23] Lassen K, Coolsen MM, Slim K (2012). Guidelines for perioperative care for pancreaticoduodenectomy: enhanced recovery after surgery (ERAS) Society recommendations. Clin Nutr.

[24] Melloul E, Hübner M, Scott M (2016). Guidelines for perioperative care for liver surgery: Enhanced Recovery After Surgery (ERAS) Society recommendations. World J Surg.

[25] Mortensen K, Nilsson M, Slim K (2014). Consensus guidelines for enhanced recovery after gastrectomy: Enhanced Recovery After Surgery (ERASVR) Society recommendations. Br J Surg.

[26] Batchelor TJP, Rasburn NJ, Abdelnour-Berchtold E (2019). Guidelines for enhanced recovery after lung surgery: recommendations of the Enhanced Recovery After Surgery (ERAS®) Society and the European Society of Thoracic Surgeons (ESTS). Eur J Cardiothorac Surg.

[27] Kehlet H, Mogensen T (1999). Hospital stay of 2 days after open sigmoidectomy with a multimodal rehabilitation programme. Br J Surg.

[28] Falcoz PE, Puyraveau M, Thomas PA (2016). Video-assisted thoracoscopic surgery versus open lobectomy for primary non-small-cell lung cancer: a propensity-matched analysis of outcome from the European Society of Thoracic Surgeon database. Eur J Cardiothorac Surg.

[29] Yan TD, Black D, Bannon PG (2009). Systematic review and meta-analysis of randomized and nonrandomized trials on safety and efficacy of video-assisted thoracic surgery lobectomy for early-stage non-small-cell lung cancer. J Clin Oncol.

[30] Bendixen M, Jørgensen OD, Kronborg C (2016). Postoperative pain and quality of life after lobectomy via video-assisted thoracoscopic surgery or anterolateral thoracotomy for early stage lung cancer: a randomised controlled trial. Lancet Oncol.

[31] Li S, Zhou K, Che G (2017). Enhanced recovery programs in lung cancer surgery: systematic review and meta-analysis of randomized controlled trials. Cancer Manag Res.

[32] Brunelli A, Thomas C, Dinesh P (2017). Enhanced recovery pathway versus standard care in patients undergoing video-assisted thoracoscopic lobectomy. J Thorac Cardiovasc Surg.

[33] Raman JD, Lin YK, Shariat SF (2017). Preoperative nomogram to predict the likelihood of complications after radical nephroureterectomy. BJU Int.

